# The Parkinsonian mimetic, 6-OHDA, impairs axonal transport in dopaminergic axons

**DOI:** 10.1186/1750-1326-9-17

**Published:** 2014-05-03

**Authors:** Xi Lu, Jeong Sook Kim-Han, Steve Harmon, Shelly E Sakiyama-Elbert, Karen L O'Malley

**Affiliations:** 1 Department of Biomedical Engineering, Washington University in Saint Louis, 1 Brookings Drive, Campus Box 1097, St. Louis, MO 63130, USA; 2 Department of Anatomy and Neurobiology, Washington University in Saint Louis, St. Louis, MO 63110, USA

**Keywords:** Neurodegeneration, Mitochondria, Microtubule, Parkinson's disease, Microfluidic devices

## Abstract

6-hydroxydopamine (6-OHDA) is one of the most commonly used toxins for modeling degeneration of dopaminergic (DA) neurons in Parkinson's disease. 6-OHDA also causes axonal degeneration, a process that appears to precede the death of DA neurons. To understand the processes involved in 6-OHDA-mediated axonal degeneration, a microdevice designed to isolate axons fluidically from cell bodies was used in conjunction with green fluorescent protein (GFP)-labeled DA neurons. Results showed that 6-OHDA quickly induced mitochondrial transport dysfunction in both DA and non-DA axons. This appeared to be a general effect on transport function since 6-OHDA also disrupted transport of synaptophysin-tagged vesicles. The effects of 6-OHDA on mitochondrial transport were blocked by the addition of the SOD1-mimetic, Mn(III)tetrakis(4-benzoic acid)porphyrin chloride (MnTBAP), as well as the anti-oxidant N-acetyl-cysteine (NAC) suggesting that free radical species played a role in this process. Temporally, microtubule disruption and autophagy occurred after transport dysfunction yet before DA cell death following 6-OHDA treatment. The results from the study suggest that ROS-mediated transport dysfunction occurs early and plays a significant role in inducing axonal degeneration in response to 6-OHDA treatment.

## Background

Genetic, imaging and environmental studies of Parkinson’s disease (PD) have revealed early problems in synaptic function and connectivity, suggesting that axonal impairment is an early, dominant feature of this disorder [1]. For example, assessment of available patient positron emission tomography data suggests that at the time of motor symptom onset there is a far greater loss of striatal dopaminergic (DA) terminals than substantia nigra DA neurons [1]. Moreover, post mortem studies show widespread axonal pathology that precedes the loss of cell bodies [2,3]. Such data support the notion that nigral neurons degenerate through a “dying back” axonopathy [4,5]. Animal models of PD-linked genes also point to axonal degeneration as an initiating factor. For example, transgenic mice expressing the PD-linked R1441G LRRK2 mutation have decreased DA terminal fields together with increased dystrophic processes and abnormal axonal swellings, findings consistent with DA axonopathy [6]. In addition, reduced axonal transport is seen with α-synuclein mutants, which accumulate in the cell soma when overexpressed in cortical neurons [7]. Emerging data also support a role in which the PD-linked genes, PINK1 and Parkin, regulate mitochondrial transport [8]. Studies in cell lines and hippocampal and cortical neurons show that PINK1 is stabilized on the outer mitochondrial membrane in response to depolarization. Stabilized PINK1 recruits Parkin, which subsequently triggers mitophagy (the autophagy of mitochondria). PD-linked mutations appear to disrupt this process allowing damaged mitochondria to accumulate and then impair axonal transport and initiate neurodegenerative processes [8].

Studies using Parkinsonian toxins also implicate mitochondrial trafficking and axon integrity in the loss of DA axons. Using specially-designed compartmented chambers and isolated axon preparations derived from transgenic GFP-tagged DA neurons, we discovered that the PD-mimetic toxin MPP^+^ rapidly (<1 h) and selectively decreased mitochondrial movement in DA axons [9,10]. In support of the notion that damaged mitochondria are re-routed to the cell body for disposal, anterograde traffic was decreased whereas retrograde trafficking was increased [10]. Temporally, following mitochondrial depolarization and immobility (30–60 min), MPP^+^ treatment led to the induction of autophagic markers such as LC3 puncta (microtubule-associated protein 1, light chain 3; also known as ATG8) [11] (3 h), and then the disruption of microtubule tracks starting at 6 h (beading) peaking between 18–24 h with extensive fragmentation [10]. Thus in MPP^+^-mediated axonal impairment, compromised mitochondria are an early event triggering downstream sequelae leading to autophagy.

6-hydroxydopamine (6-OHDA) is another widely used Parkinsonian toxin that induces degeneration of DA neurons [12]. 6-OHDA has been shown to disrupt complex I of the mitochondrial electron transport chain and increase generation of reactive oxygen species (ROS) that contributes to an apoptotic form of cell death. However, it is not known how 6-OHDA induces axonal damage.

Using our newly described compartmented microdevices [9] we studied the effects of 6-OHDA on various processes using murine mesencephalic cultures. Here we show that 6-OHDA decreases mitochondrial and vesicular movement in DA axons and explore potential mechanisms underlying these effects.

## Materials and methods

### Cell culture

**Figure 1:**
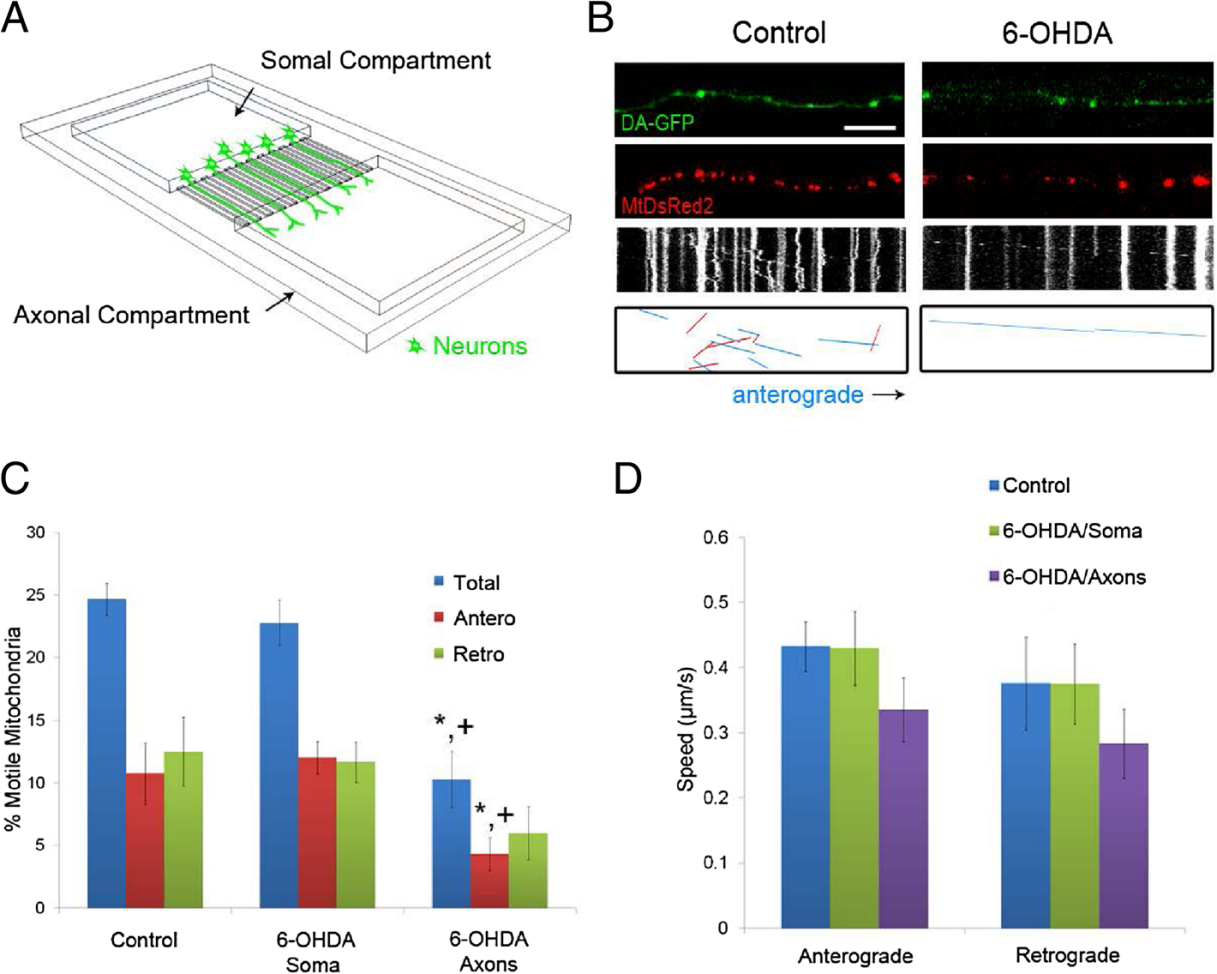
**6-OHDA rapidly decreases mitochondrial movement in DA axons. A)** Diagram of microdevice **B)** Axonal movement of mitochondria in control and 6-OHDA treated axons. DA-GFP cultures (Top panels) grown in microdevices and transduced with MitoDsRed2 (Middle panels) were imaged 30 minutes after treatment with 6-OHDA. Resulting kymographs are shown below. For additional clarity tracks of moving particles are depicted in the bottom panels: blue lines denote anterograde movement and red lines indicate retrograde trafficking. Scale bar indicates 10 μm. Quantification of **C)** moving mitochondria (n = 4–5 devices per group with 4–5 axons analyzed per device) and **D)** mitochondrial speeds. The latter were calculated as described [10] (n = 60–80 mitochondria per group). In **C** and **D**, data are represented as mean ± SEM, * + indicates p < 0.05 versus control and 6-OHDA in somal compartment.

Microdevice fabrication and cell culture were performed as previously described [9,10]. The width of the microchannels for the microdevice (Figure 1A) was decreased to 5 μm from 10 μm to increase the probability of observing singly labeled axons and to limit axonal bundling. Other dimensions of the microdevice were unchanged from those previously reported. Midbrain tissues were harvested from E14 Tg(TH-EGFP) DJ76GSAT transgenic mouse embryos (Jackson Laboratories, Bar Harbor, ME). Animal procedures were performed in accordance with the National Institutes of Health Guide for the Care and Use of Laboratory Animals. All GFP positive tissues were pooled. For seeding, 60,000 cells were plated per somal compartment in DMEM/F12 (Invitrogen, Carlsbad, CA) with 10% FBS (Invitrogen) supplemented with 1× B-27 (Invitrogen) and 100 I.U. penicillin/100 μg/mL streptomycin (CellGro, Manassas, VA). Cells were concentrated via centrifugation to obtain a final loading volume of 5 μL. Cells were fed with fresh Neurobasal media (Invitrogen) and supplemented with 0.5 mM glutamine (Sigma-Aldrich, St. Louis, MO) and 1× B27 every other day. On DIV 5, the media was also supplemented with AraC (Sigma-Aldrich, final concentration: 5 μM) to limit glial proliferation. Netrin I (300 ng/mL, R&D Systems, Minneapolis, MN) was added into the axonal compartment as a chemo-attractant. Addition of toxin is as follows: from an initial stock of 6-OHDA (Sigma-Aldrich), serial dilutions were performed using deoxygenated water to a volume of 100 μL (per compartment) for a final concentration of 40 (for assessing autophagy) or 60 μM, which was used for all other experiments.

### Mitochondrial and synaptic vesicle labeling

Transport was assessed on DIV 12 or 13 by adding 6-OHDA to both or either axonal/somal compartment. To label mitochondria, a plasmid containing mitochondrially-targeted DsRed2 was generated by inserting a mitochondrial targeting sequence (MLSLRQSIRFFK, the signal peptide of COX IV) in front of DsRed2 (Clontech, Mountain View, CA). The mitoDsRed2 was then subcloned into a FUGW lentiviral expression vector provided by Dr. Jeffrey Milbrandt (Washington University in St. Louis). The lentivirus was generated in HEK293T cells using procedures previously described [13]. Cells were transduced with the virus on DIV 2 for 5–6 hours. By limiting viral transduction to obtain 60-70% labeling efficiency, many more singly labeled axons per microchannel were observed. A lentivirus for labeling synaptic vesicles was generated using a plasmid containing synaptophysin fused in frame with cerulean (provided by Dr. Rachel Wong, University of Washington Seattle).

### Microtubule structure

The integrity of microtubules was assessed by immunostaining with antibodies against acetylated tubulin (AcTub; Sigma-Aldrich) and tyrosine hydroxylase (TH) (Pel-Freeze Biological, Rogers, AR) after treatment with 6-OHDA in the axonal compartment. Axons with three AcTub breaks or more were considered damaged and the number as a percentage of total axons in TH-positive and negative axons was determined.

### Retrograde degeneration study

On DIV 13, the axonal compartment was treated with 6-OHDA and then cell death was assayed by labeling with propidium iodide (1 μg/mL, Sigma-Aldrich) at 24 and 48 hours. Fluorescent and bright field images were taken of cell bodies within 350 μm of the microchannel opening in the somal compartment. Cell death was quantified by calculating the fraction of propidium iodide positive cells.

### Autophagy

On DIV 5–6, cells were transfected with a GFP-tagged LC3 expression vector provided by Dr. Chris Weihl [14]. 24 hours after transfection, cells were treated with 6-OHDA for the specified time, fixed, and stained with antibodies against tyrosine hydroxylase (TH) (Pel-Freeze Biological, Rogers, AR). Cells with LC3-GFP puncta were counted and compared to the total number of LC3-GFP positive cells in TH-positive and negative ones.

### Confocal imaging

Time lapse images of mitochondrial movement were taken using a Zeiss LSM510 Meta NLO Multiphoton System (Carl Zeiss, USA) on Axiovert 200 M inverted microscope with a 40× water objective [C-Apochromat 40×/1.2 W Corr.1.2 numerical aperture, collar correction (0.14-0.18)]. The microscope contains a heated stage which includes a Pecon CTI-Controller 3700 for regulating 5% CO_2_ (Zeiss, USA) and a Pecon TempControl 37–2 digital (Zeiss) for heating the stage to 37°C for the duration of the image recordings. A total of sixty images at 5 s intervals (mitochondria and vesicles) or 180 images at 2 sec intervals (vesicles) were recorded and then used to generate kymographs for measurement of transport. Filters used for visualizing the fluorescent markers included a 488 nm argon laser and 505 nm long pass emission filter (GFP), 543 nm HeNe laser and 560 nm long pass emission filter (MitoDsRed2) and 458 nm argon laser and 466–514 meta emission filter (Syn-Cer).

### Kymograph analysis of moving particles

Kymographs generated using Image J (NIH, Bethesda, MD) were analyzed as described previously [10]. Time lapse images were imported into ImageJ and then the image was split into individual channels. A threshold image of the mitochondrial channel was used for analysis. A segmented line was then used to select the region of interest. An add-on to ImageJ called Multiple Kymographs was then used to generate each kymograph derived from the region of interest. Each diagonal line upon a kymograph represented a moving particle while the straight lines represented nonmoving particles. The angle and length of each line was then used to calculate the direction and speed of the moving mitochondria [10].

### Mitochondrial membrane potential and size

Cells were loaded with 25 nM of Tetramethylrhodamine ethyl-ester (TMRE, Invitrogen) for 25 minutes prior to imaging. Changes in mitochondrial membrane potential were determined by differences in TMRE membrane potential along an axonal region of interest before and after treatment with 6-OHDA [15]. Mitochondrial cross sectional area was estimated by mitoDsRed2 fluorescence using Image J’s particle analysis.

### Statistical analysis

Statistical analysis was performed using Statistica (Statsoft, Tulsa, OK). One way ANOVA, followed by Scheffe's F test or Student’s t-test were used to determine statistical significance. P values below 0.05 were determined to be statistically significant.

## Results

### Mitochondrial movement decreased in DA and non-DA axons

To investigate how 6-OHDA influences axonal mitochondrial transport, we used a microdevice to isolate the axons and labeled the mitochondria using a lentivirus expressing mitochondrially targeted DsRed2 to allow visualization in live cells. Initial dose response experiments using cultured DA neurons suggested that 60 μM 6-OHDA led to ~60% cell death after 24 h [16]. Using this dose, there was a 50% decrease in DA mitochondrial motility 30 minutes after 6-OHDA treatment in the axonal compartment (Figure 1B,C). Taking advantage of the fluidic isolation between the somal and axonal compartment, experiments were performed where only the somal compartment was treated with 6-OHDA to determine whether there was an anterograde effect on axonal mitochondrial transport. After 30 minutes, DA mitochondrial motility or movement speed within the microchannels showed no statistically significant change compared to vehicle-treated controls (Figure 1C,D). Finally, of the mitochondria that were still motile, there were no significant differences in transport speed in either an anterograde or retrograde direction (Figure 1D).

**Figure 2:**
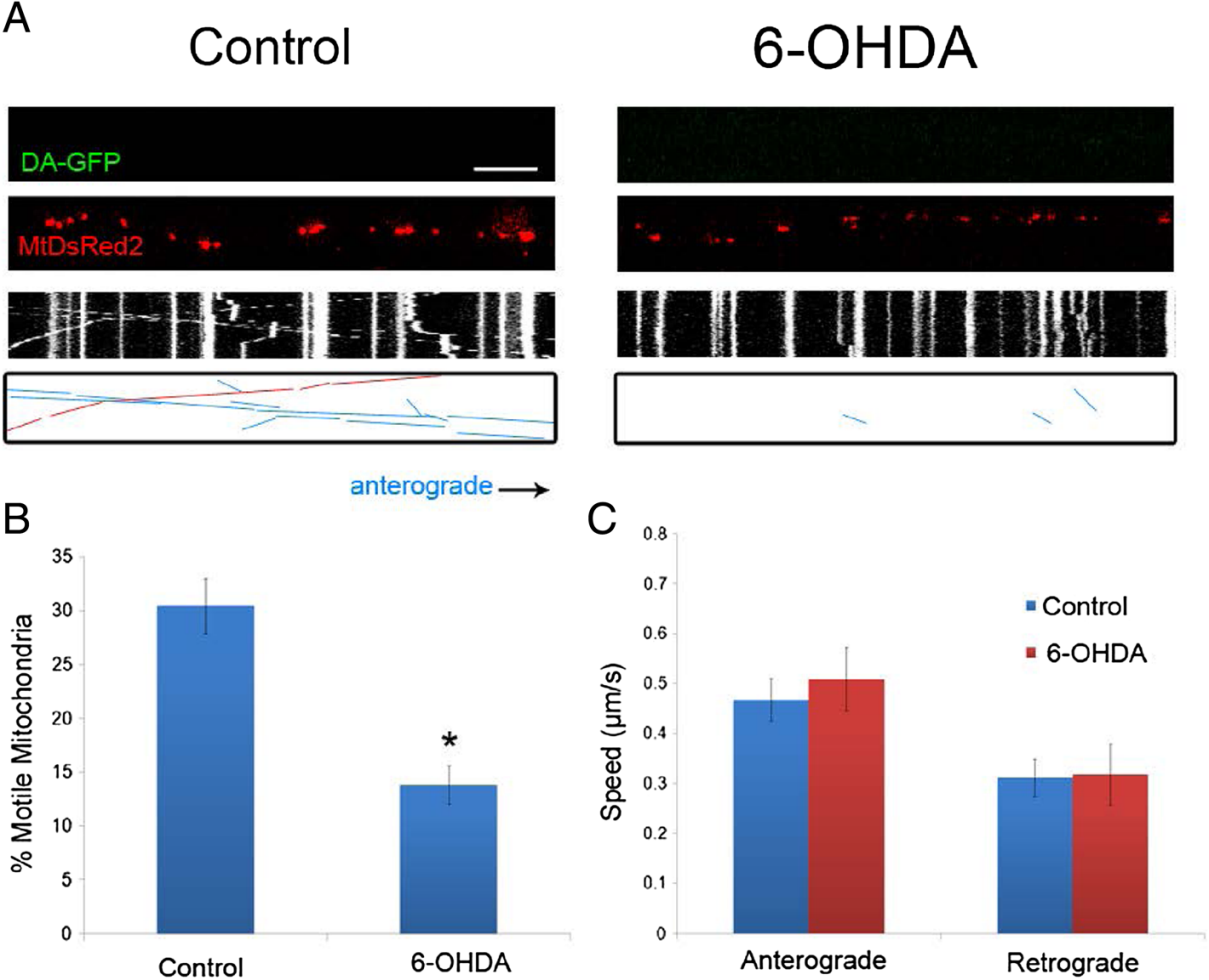
**6-OHDA rapidly decreases mitochondrial movement in non-DA axons. A)** Axonal movement of mitochondria in control and 6-OHDA treated axons. Non-GFP positive axons (non-DA; Top panels) that were labeled with MitoDsRed2 (Middle panels) were selected for imaging 30 minutes after treatment with 6-OHDA. Resulting kymographs are shown below. For additional clarity tracks of moving particles are depicted in the bottom panels: blue lines denote anterograde movement and red lines indicate retrograde trafficking. Scale bar indicates 10 μm. Quantification of **B)** moving mitochondria in both anterograde and retrograde directions (n = 3–4 devices per group from with 3–5 axons analyzed per device) and **C)** mitochondrial speeds of motile mitochondria. The latter were calculated as described [10] (n = 90–120 mitochondria per group). In B and C, data are represented as mean ± SEM, *: indicate p < 0.05 versus control.

Because 6-OHDA is easily oxidized *in vitro* to p-quinones and ROS species such as hydrogen peroxide, 6-OHDA may exert its toxic effect via an extracellular mechanism without the need for uptake via the dopamine transporter [17]. In fact, we have previously shown that even small doses and short time treatments with 6-OHDA lead to death of DA and non-DA neurons in culture [16]. Not surprisingly then, mitochondrial transport in non-DA axons was also significantly decreased in terms of total mitochondrial motility without an effect on anterograde or retrograde velocities (Figure 2). Taken together, 6-OHDA led to a 50% decrease in mitochondrial motility 30 min after treatment in both DA and non-DA axons.

### 6-OHDA decreases mitochondrial membrane potential but does not affect mitochondrial size

**Figure 3:**
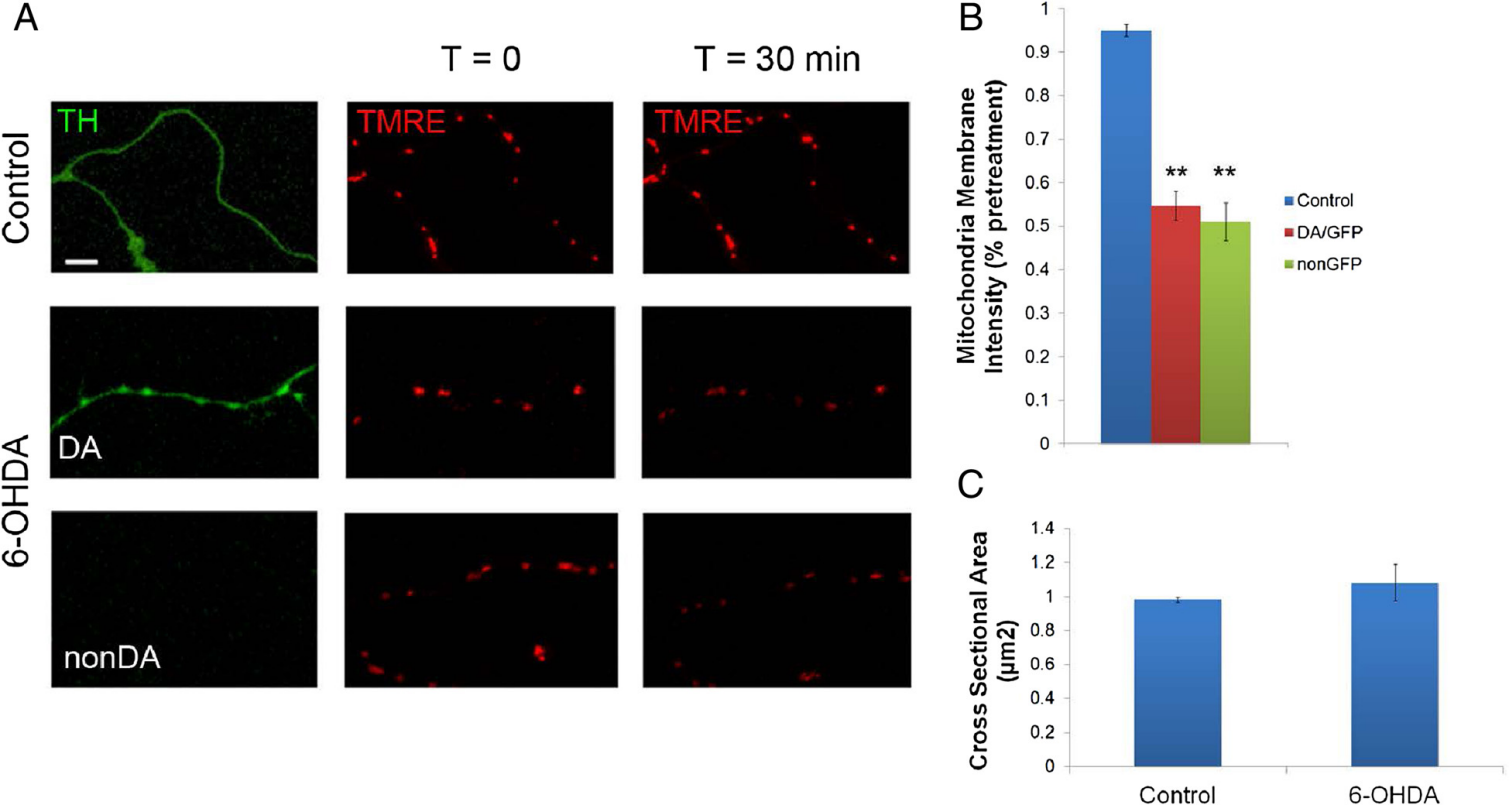
**6-OHDA rapidly depolarizes mitochondria in both DA and non-DA axons. A)** To ensure rapid, even labeling of mitochondria with TMRE (25 nM), axons were assessed after they had exited the microdevice channels. Scale bar indicates 10 μm. **B)** 6-OHDA significantly decreased mitochondrial membrane potential (ΔΨm) in DA and non-DA axons. Data indicate mean ± SEM from four independent experiments (n = 18–30 axons per group). ** indicates p < 0.001 versus control. **C)** Quantification of cross-sectional area of DA mitochondria before and after treatment with 6-OHDA. Data indicate mean ± SEM.

Mitochondrial membrane potential is a commonly used parameter for determining mitochondrial health and may act as a signal to regulatory machinery that could lead to cessation of mitochondrial movement. Therefore to assess relative changes in mitochondrial membrane potential, we assessed the ability of mitochondria to accumulate a membrane voltage sensitive dye, TMRE, and determined membrane depolarization by a decrease in TMRE fluorescent intensity. Thirty minutes after treatment with 6-OHDA, a significant decrease in TMRE fluorescence was observed in both DA-GFP axonal mitochondria and non-GFP mitochondria (Figure 3A,B). To determine whether mitochondrial fragmentation plays a role in cessation of movement, mitochondrial cross-sectional area was measured using the Image J particle analysis program. As TMRE fluorescence is lost upon membrane depolarization, it cannot be used to accurately measure changes in relative mitochondrial morphology. Instead, mitoDsRed2 was used to measure mitochondrial size. Even after 1 hour of 6-OHDA treatment there was no significant difference between cross-sectional areas of the control and toxin-treated groups (Figure 3C).

### 6-OHDA decreases axonal transport of synaptic vesicles

**Figure 4:**
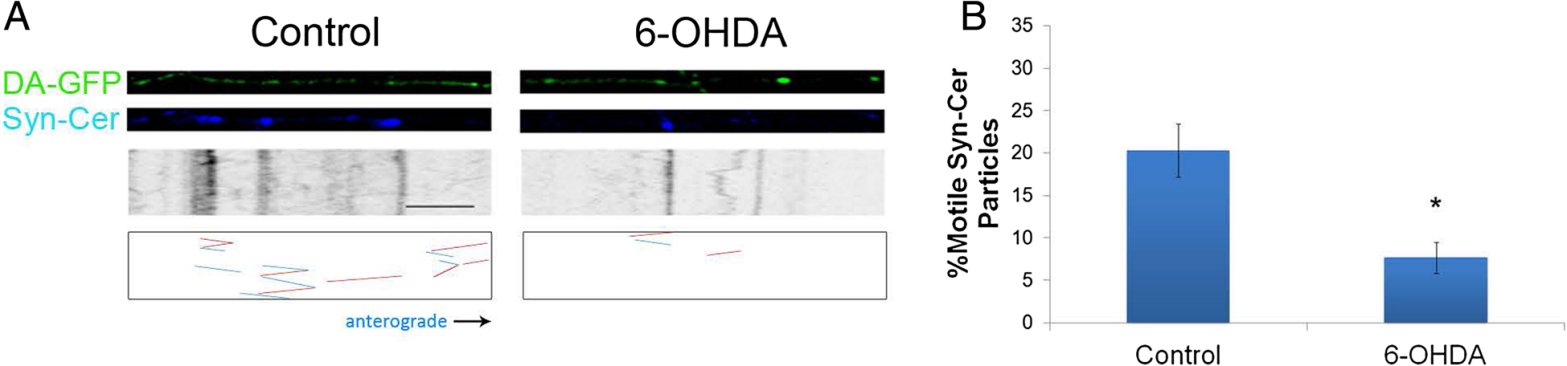
**6-OHDA also decreases synaptic vesicle movement in DA axons. A)** DA-GFP cultures (Top panels) in microdevices were transduced with Syn-Cer lentivirus (Middle panels) at DIV2. Vesicular movement was assessed on DIV12–13 before and after toxin treatment. Resulting kymographs are shown below. Because of the smaller size of vesicular particles and the relative “dimness” of the cerulean emission, tracks of moving particles are shown in bottom panels for clarity. Red represents retrograde movement whereas blue is anterograde trafficking. **B)** Quantification of moving vesicles was determined as described in Materials and Methods. Scale bar: 5 μm. Mean ± SEM, total of 8 (control) and 8 (6-OHDA-treated) axons from 5–7 devices per group. * indicates p < 0.05 versus control.

Mitochondria are not the only cargo being transported along the axon. Using standard bright-field microscopy, it is common to see many particles moving bi-directionally along the axon. However, when assessing particle movement in our microchannels, the particles tend to blend into the shadow of the microchannels, as axons adhere to the channel sides, hence particle movement cannot be measured using a standard bright-field microscopy. Therefore, to determine whether 6-OHDA specifically disrupts mitochondrial transport or whether it may affect transport of other axonal cargo, movement of synaptic vesicles was assessed with a synaptophysin-cerulean marker. Previous reports from this lab showed that synaptophysin-cerulean marked small rapidly moving vesicles that did not co-localize with mitochondria [10]. Similar to the decrease in mitochondrial motility, after 30 minutes of treatment with 6-OHDA the movement of synaptic vesicles in both the anterograde and retrograde direction was reduced by 60-70% (Figure 4). Due to the low number of moving particles, meaningful velocity data could not be obtained from measuring the remaining motile particles. These findings show that 6-OHDA affects axon transport machinery resulting in decreased axonal transport of two important cargoes, synaptic vesicles and mitochondria.

### 6-OHDA damages microtubule tracks after 6 hours and induces retrograde degeneration

**Figure 5:**
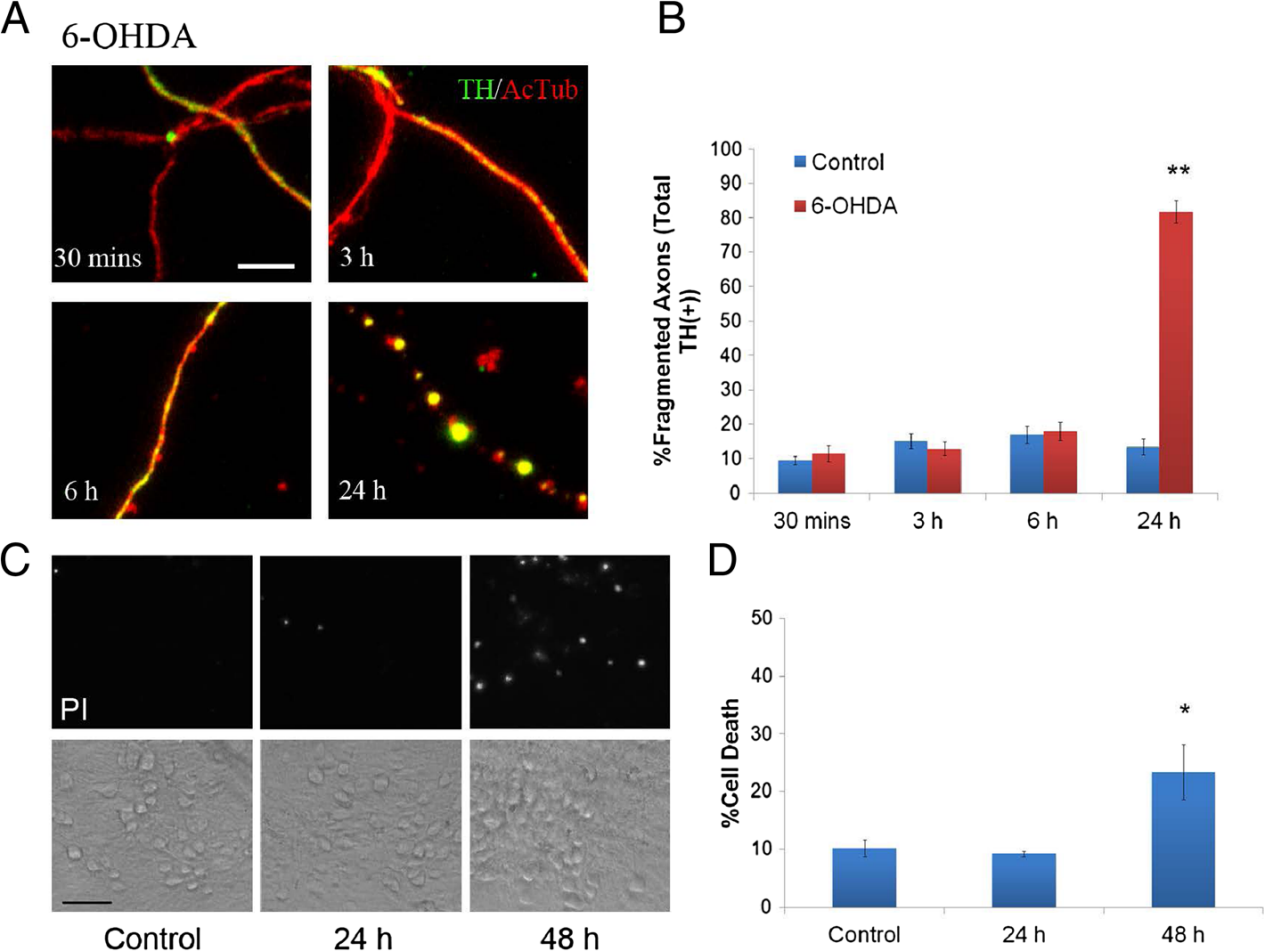
**6-OHDA induces microtubule disruption and retrograde degeneration in vitro. A)** Integrity of microtubule tracks was assessed by measuring tubulin fragmentation in DA cultures treated with 6-OHDA at the indicated times and then fixed and stained with antibodies against AcTub and TH. Significant fragmentation of AcTub is not seen prior to 6 hours, but is readily apparent at 24 hours. **B)** TH positive axons with fragmented AcTub staining were quantified. One hundred to three hundred DA-GFP axons were counted per dish and 4–5 dishes were used per group. Scale bars indicate 10 μm. Bars represent mean ± SEM. ** indicates p < 0.001 versus control. **C)** The axonal side of cultures grown in microdevices were treated with 6-OHDA for 24 or 48 hours. Subsequently, the cell body compartment was treated with 1 μg/ml of propidium iodide (PI) and then imaged 30 minutes later to assess cellular degeneration. Note very little PI staining is seen in control (48 hours without 6-OHDA treatment) or 24 hour after 6-OHDA cultures. **D)** Quantification of cell death using propidium iodide. n = 4 devices per group. Scale bar indicates 40 μm. Data are represented as mean ± SEM, *indicates p < 0.05 versus control.

Destabilization of the cytoskeleton tracks along which transport occurs could potentially be a causative factor for the disruption of organelle and vesicular movement along the axon. Microtubules are the primary tracks along which axonal transport occurs. Therefore to assess microtubule integrity, we stained for acetylated tubulin (AcTub), a marker associated with stabilized microtubules. Control axons showed smooth and continuous AcTub staining at all time points whereas axons treated with 6-OHDA only remained intact for about 6 hours (Figure 5A,B). By 24 hours, more than 80% of DA (Figure 5B) and non-DA axons (83 ± 4%) showed a significant number of breaks and fragmentation in AcTub staining. It suggests that microtubule destabilization is not a main causing factor for the early effect of 6-OHDA on axonal transport.

*In vivo*, 6-OHDA induces retrograde degeneration of DA neurons following injection within the striatum [18,19]. Normally, whether this occurs *in vitro* is impossible to assess given the difficulty in assigning processes to cell bodies, however, this is readily done in the compartmented chambers. Thus, to assess whether this form of retrograde degeneration also occurs *in vitro* and determine the time course for when it occurs, 6-OHDA was applied only to the axonal chamber and cell death was assayed using propidium iodide at 24 and 48 hours post treatment. While the majority of axons showed fragmentation of acetylated microtubules at 24 hours (Figure 5A,B), no significant cell death was detected at this time in the somal compartment near the microchannels. A significant increase in cell death was only measured 48 hours after 6-OHDA treatment (Figure 5C,D). These results confirm those shown *in vivo* and highlight the utility of the microdevice system to model and study retrograde neuronal degeneration.

### 6-OHDA induces autophagosome formation

**Figure 6:**
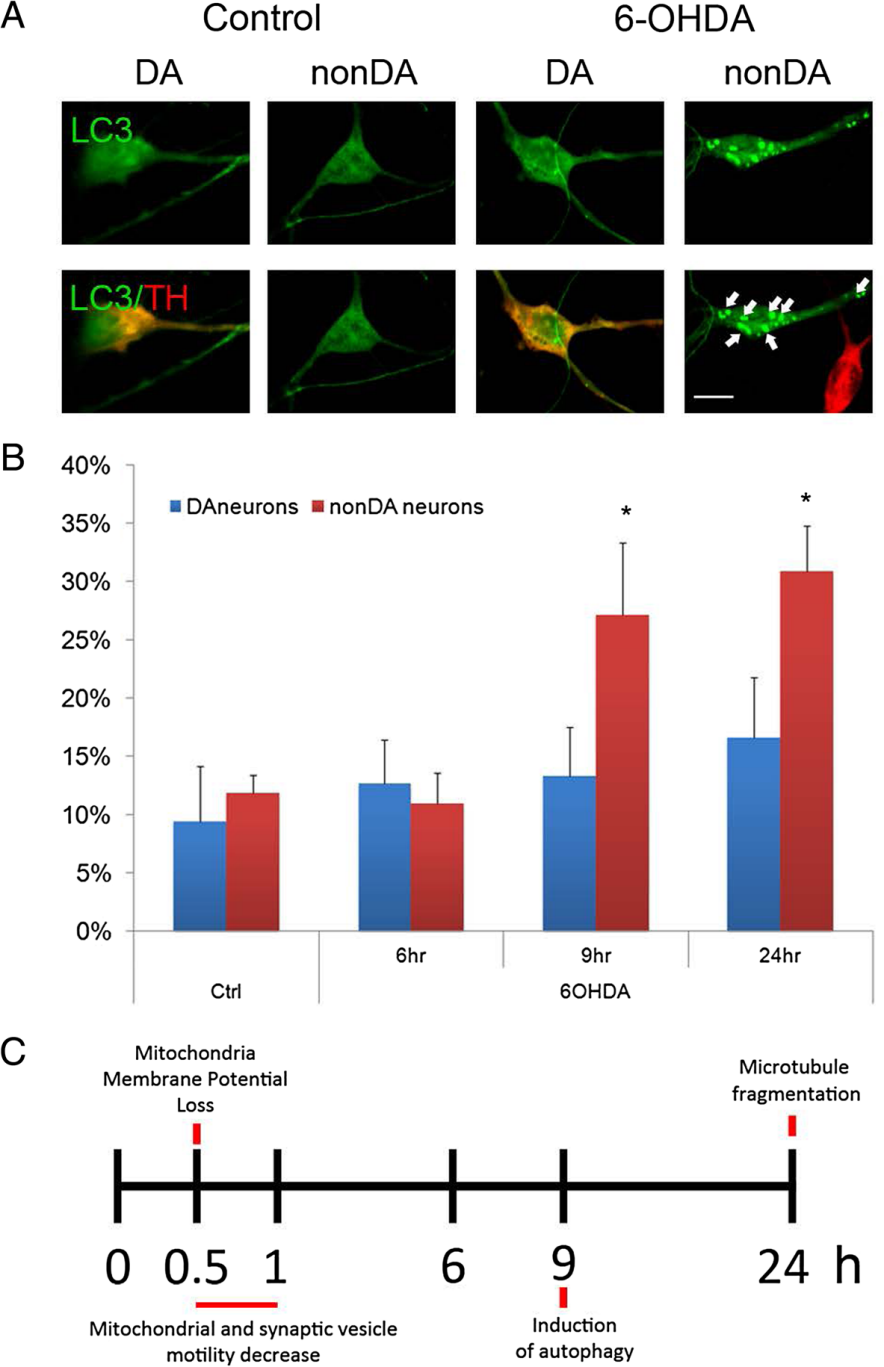
**Autophagy precedes cell death in midbrain neurons following 6-OHDA treatment. A)** Autophagy was assessed by introducing a GFP-tagged LC3 expression clone at DIV6 and treating midbrain cultures 1 d later with 6-OHDA. LC3-positive puncta (arrows) were assessed by GFP fluorescence in representative neurons in control and after toxin treatment. **B)** The number of cells with at least three LC3-GFP puncta were counted and expressed as percentage of all neurons that were LC3-GFP positive, regardless of whether the LC3-GFP signal in these neurons was diffuse or punctated. Scale bar indicates 10 μm. Mean ± SEM from 3 independent experiments (n = 3–5 per group), *p < 0.05 versus control. **C)** Timeline of 6-OHDA induced events.

Damaged mitochondria can be harmful and degraded by a form of autophagy known as mitophagy. Successful removal of damaged mitochondria could be critical for maintaining axonal health and limiting secondary damage. Improper regulation of the mitophagy process could adversely affect neuronal health. Previously, 6-OHDA has been shown to induce autophagy in rat models [19] and cell lines [20]. To determine whether 6-OHDA could also induce autophagy and whether it could be a cause for mitochondrial movement in axons from murine mesencephalic neurons *in vitro*, the appearance of LC3, an autophagy marker, was assessed. Under control conditions, LC3-GFP exhibited a continuous fluorescence within the cytosol. However, 9 hours after 6-OHDA treatment, LC3 fluorescence took on a punctate appearance thought to represent its aggregation on membranes of autophagosomes (Figure 6A,B). There was a significant increase in the percentage of LC3-GFP positive puncta in nonDA neurons with only a trend toward increased positive puncta in DA neurons, suggesting distinctive roles of autophagy in the 6-OHDA model. Also, it appears that the formation of autophagosomes is a later event, which occurs after disruptions in axonal transport.

### NAC and MnTBAP rescue mitochondrial transport

6-OHDA has been shown to inhibit mitochondrial complex I activity [21] and has been suggested to induce cell death via oxidative stress primarily by increased ROS formation [12]. It has also been found that ROS scavengers were potent in protecting cell bodies against the toxic oxidative byproducts of 6-OHDA [22]. To investigate whether oxidative stress induced by ROS formation also plays a role in disrupting axonal transport of mitochondria, we investigated whether anti-oxidants such as NAC and MnTBAP could rescue this early event in axonal degeneration. In addition, we also investigated whether EGTA could rescue mitochondrial transport disruption since calcium signaling plays an important role in axon degeneration [23]. Consistent with the notion that blocking ROS prevents subsequent impairment of mitochondrial processes [24], both NAC and MnTBAP protected DA mitochondria from transport impairment after treatment with 6-OHDA (Table 1). NAC also rescued synaptic vesicle motility (vesicle motility: 23.8 ± 2% compared to 6-OHDA: 7.6 ± 1.2%, p < 0.05). In contrast, EGTA did not protect against the loss of mitochondrial mobility suggesting that calcium did not play a role in this injury, at least at early time points (Table 1).

**Table 1:** Effects of antioxidants and calcium chelation on 6-OHDA-disrupted DA mitochondrial transport

	**Motile Mitochondria**
**Control**	24.6 ± 1.3%*
**6-OHDA**	10.3 ± 2.2%
**+NAC**	25.7 ± 3.3%*
**+MnTBAP**	28.2 ± 6.5%*
**+EGTA**	8.34 ± 3.9%

Data indicates mean ± SEM. * indicate p < 0.05 versus 6-OHDA. [NAC] = 2.5 mM, [MnTBAP] = 100 μM, [EGTA] = 2.5 mM.

## Discussion

The use of novel microdevices to isolate axons from cell bodies combined with real time imaging of axonal mitochondria and synaptic vesicles provided new insights into the temporal sequence of cellular changes underlying 6-OHDA-mediated dysfunction (Figure 6C). The present findings demonstrated that (1) 6-OHDA rapidly blocked (<30 min) mitochondrial trafficking in DA axons, a process accompanied by a loss in mitochondrial membrane potential; (2) the effects of 6-OHDA *in vitro* were not selective for DA mitochondria as non-DA mitochondria were equally affected; (3) remaining motile mitochondria exhibited decreased movements in anterograde direction; (4) 6-OHDA also decreased axonal transport of synaptic vesicles within 30 min; (5) both mitochondrial and vesicular transport could be rescued by pre-treatment with anti-oxidants, such as NAC; (6) 6-OHDA affected microtubule tracks in axons 6–9 hr after axonal transport ceased and death was observed in cell bodies after 48 hours. (7) 6-OHDA caused the formation of autophagosomes after 9 hr of treatment. Taken together these data demonstrate that 6-OHDA induces cell death via a retrograde dying back process that can be blocked by free radical scavengers.

Widely used as an animal model of PD, 6-OHDA quickly oxidizes to form a variety of free radical species which can lead to toxic sequelae, such as DNA damage [25] and oxidation of proteins [26-28]. Although oxidative protein damage leads to ER stress and the upregulation of the unfolded protein response [29,30], this appears to serve as a protective measure in DA neurons [25]. Instead, DNA damage leads to activation of a p53- and Puma-dependent apoptotic cascade *in vivo* and *in vitro*; loss of p53 and Puma rescues 6-OHDA-mediated cell death [25,31,32].

How might these studies fit with early organellar transport impairment, retrograde dying back and loss of axonal integrity? Interestingly, *in vivo* studies using 6-OHDA to damage the nigrostriatal projection showed that activation of the Akt/mTOR pathway could block apoptosis, preserve DA cell bodies, prevent autophagy and suppress retrograde axon degeneration [19]. Mechanistically, these data underscore the importance of preserving axonal function. The present *in vitro* findings further emphasize very early events that occur in the axonal compartment that set the stage for later events including the loss of connectivity and ultimately cell death. It should be stressed that the direction of degeneration is also an important caveat and differences may exist between anterograde and retrograde models of degeneration, particularly for degeneration in the nigrostriatal region. For example while many *Wlds* studies have shown that it delays and protects against axonal loss in anterograde degeneration, it does not confer axonal protection against retrograde degeneration [33-35]. The model and findings of this study are then directly relevant to understanding the retrograde dying back nature of Parkinson's and other neurodegenerative diseases. Akin to the *in vivo* results, inclusion of toxin in the somal compartment did not immediately lead to anterograde loss of axonal transport (Figure 1C) whereas axonal transport was rapidly compromised in the retrograde direction (Figure 1). Although we have not yet tested the role of Akt/mTOR, we would predict that these cascades are downstream of ROS generation given the timing by which autophagy is stimulated (9 h; Figure 6) and that microtubules exhibit fragmentation (24 h; Figure 5).

Because the anti-oxidants NAC and SOD1 mimetics rescued 6-OHDA-immobilized mitochondria, it is likely that axonal transport dysfunction and degeneration is due to the increased generation of ROS species affecting general transport processes. The latter might include oxidation of the transport proteins themselves or oxidation of an adaptor protein responsible for connecting the motor protein to the organelle. For example, impairment of motor proteins such as kinesin-1disrupts axonal transport and induces axonal degeneration [36]. Adaptor proteins such as Miro and Milton can be oxidized but are also regulated by calcium changes that can affect their binding to each other. Given the lack of effect of EGTA (Table 1) and previous experiments showing no change in calcium levels in response to 6-OHDA [26], that makes this hypothesis less likely to be correct. Alternatively, 6-OHDA-generated ROS might block mitochondrial ATP production leading to a loss of energy required by the motor proteins to function [37]. Consistent with this notion, a recent report showed that hydrogen peroxide led to the loss of mitochondrial transport in hippocampal neurons, an effect mimicked by blocking ATP synthesis [38]. Previously we showed that this was not the case in DA axons treated with another widely used PD-mimetic, MPP^+^[10]. Surprisingly, despite being a Complex I inhibitor, MPP^+^ also rapidly blocked mitochondrial transport via a redox sensitive process and not via ATP loss [10]. The extent to which ATP deficiency mediates 6-OHDA effects in the trafficking of mitochondria remains to be tested.

Although 6-OHDA and MPP^+^ are often lumped together as PD-mimetics, their effects on neurons and in particular DA neurons are quite unique. Although both toxins lead to the death of DA neurons in a protein synthesis-, p53-, and PUMA-dependent manner [16,25,29,39], the downstream signaling pathways diverge in many ways [40]. In terms of axonal impairment, 6-OHDA and MPP^+^ both lead to the loss of neurites prior to cell body death [10,16,40,41] as well as mitochondrial dysfunction and loss of motility in DA axons. In contrast to 6-OHDA, MPP^+^ exhibits a more specific effect on mitochondrial movement that cannot be rescued by ROS scavengers, such as MnTBAP (SOD mimetic); MPP^+^ could exert its toxicity by disrupting the redox state (e.g. generation of glutathione or hydrogen peroxide) of the mitochondria after internalization whereas 6-OHDA could directly auto-oxidize to ROS, such as hydrogen peroxide both inside and outside of a cell [10]. The present findings show that 6-OHDA-generated ROS affects many axonal transport processes including mitochondrial and synaptic vesicle trafficking. Taken together, these data further emphasize that 6-OHDA and MPP^+^ impair axons and cell bodies by distinct cellular mechanisms.

The PD-linked genes, Pink1 and Parkin appear to play important roles in regulating mitochondrial dynamics such as movement and morphology as well as mitochondrial removal after damage [42-45]. Many studies especially in neuroblastoma cells show that mitochondrial membrane depolarization stabilizes Pink1 on the outer mitochondrial membrane leading to the recruitment of Parkin, cessation of movement and the rapid induction of autophagy [46]. Previously we showed that MPP^+^ depolarized DA mitochondria and blocked trafficking within 1 hr following treatment; autophagy was observed shortly thereafter (3 hr; [10]). Despite the rapid depolarization and cessation of mitochondrial movement in 6-OHDA-treated axons, autophagy was observed after 9 hrs (Figure 6). It is unclear why this delay for non-DA neurons or even less for DA neurons exists since damaged mitochondria could serve as a source for leaking ROS that can further exacerbate the oxidative damage to the axon. The role of autophagy in 6-OHDA has been inconsistent in the literature [47,48]; one study showed that blocking autophagy helped protect SH-SY5Y cells against 6-OHDA toxicity, whereas the other study showed that regulation of 6-OHDA induced autophagy had no effect on the death of SK-N-SH cells derived from SH-SY5Y cells, a human neuroblastoma cell line. Although not significant, there was a clear trend towards autophagosome formation in DA neurons. Also, we noted differences in the appearance of LC3 puncta between DA and nonDA neurons, which calls for further investigation to determine the characteristics of autophagy in primary DA neurons.

Many additional questions must be addressed, such as could ROS generated from mitochondrial damage or 6-OHDA oxidation limit intra-axonal recruitment of Pink1 to the mitochondria or its stabilization? Perhaps, as suggested above, it is a loss of ATP that impairs organelle movement and Pink1/Parkin are only involved at later time points if at all. Other pathways exist that trigger autophagy, and it may be that these represent alternative, yet slower mechanisms to ensure axonal removal of damaged mitochondria or vesicles [49,50]. In any case, the delay in the onset of autophagy suggests that damaged mitochondria are remaining within the axons and are not being removed which may contribute to further axonal impairment due to steric hindrance. Moreover, just the appearance of LC3 puncta is not indicative of the successful removal of damaged organelles, since the formation of an autolysosome is required for complete removal of damaged mitochondria. Excessive autophagosome formation without proper trafficking could also lead to transport blocks.

It is clear that axonal transport disruptions play an early and important role in 6-OHDA induced axonal degeneration. While differences exist between 6-OHDA’s and MPP^+^’s effects on axonal transport, the observation that these two widely used toxin models converge on early dysregulation of mitochondrial transport prior to other events such as microtubule fragmentation points to the importance of maintaining the health of the axonal compartment. While it remains to be seen whether other PD toxin models, such as paraquat or rotenone induce similar patterns of axonal impairment in midbrain DA axons, maintenance of mitochondrial transport could bridge the gap between different causes of axonal degeneration and suggest a common therapeutic strategy. Improper trafficking of vital organelles, such as mitochondria and other signaling vesicles may lead to energy deficits, exacerbate oxidative stress, ionic disruption, accumulation of misfolded proteins, or the inability of retrograde signaling molecules to reach their somal targets. All of these processes could lead to the activation of axonal death pathways. The discovery of Sarm1, a protein required for the activation of injury-induced axonal degeneration points to the existence of one such axonal death signaling pathway [51]. Whether Sarm1 or an axon regenerative pathway, such as mTOR [52,53], is applicable to axonal impairment in PD remains to be addressed. The development of microdevices provides a tool to rigorously characterize cell populations such as neurons whose extended, compartmented morphology renders previously intractable problems solvable. These new technologies continue to enhance and expand the available toolset for understanding key biological processes in order to develop better therapies for patients suffering from major neurological disorders.

## Conclusions

Using a microplatform, we showed that 6-OHDA, one of the most commonly used parkinsonian mimetics, disrupts the motility of mitochondria and synaptic vesicles in DA axons early in the process of axonal degeneration. In addition, local exposure of axons to 6-OHDA was enough to induce axonal loss and eventually, cell death. The rescue of 6-OHDA induced mitochondrial transport dysfunction by anti-oxidants suggests that ROS or disruption of cellular defenses against ROS may contribute significantly to the dying-back form of degeneration seen in Parkinson's disease.

## Abbreviations

6-OHDA: 6-hydroxydopamine; PD: Parkinson's disease; DA: Dopaminergic; GFP: Green fluorescent protein; NAC: N-acetyl-cysteine; MnTBAP: Mn(III)tetrakis(4-benzoic acid)porphyrin chloride; EGTA: Ethylene glycol tetraacetic acid; TH: Tyrosine hydroxylase; AcTub: Acetylated tubulin; TMRE: Tetramethylrhodamine ethyl-ester; ROS: Reactive oxygen species; DIV: Day in vitro; FBS: Fetal bovine serum.

## Competing interest

The authors declare that they have no competing interests.

## Authors’ contributions

XL, JSK, KOM, and SSE were involved in the design of experiments. SH performed all animal procedures. XL and JSK performed experiments and data analysis, while XL drafted the manuscript. All authors participated in revising, editing and approving the final manuscript.
